# Sensitivity to model structure: a comparison of compartmental models in epidemiology

**DOI:** 10.1057/hs.2015.2

**Published:** 2017-12-19

**Authors:** Sheetal Prakash Silal, Francesca Little, Karen I. Barnes, Lisa Jane White

**Affiliations:** 1 Department of Statistical Sciences, University of Cape Town, Cape Town, South Africa; 2 Department of Medicine, University of Cape Town, Cape Town, South Africa; 3 Mahidol-Oxford Tropical Medicine Research Unit, Mahidol University, Bangkok, Thailand; 4 Nuffield Department of Clinical Medicine, Churchill hospital, University of Oxford, Oxford, UK

**Keywords:** mathematical modelling, differential equations, sensitivity testing

## Abstract

Compartmental models have provided a framework for understanding disease transmission dynamics for over 100 years. The predictions from these models are often policy relevant and need to be robust to model assumptions, parameter values and model structure. A selection of compartmental models with the same parameter values but different model structures (ranging from simple structures to complex ones) were compared in the absence and presence of several policy interventions to assess sensitivity to model structure. Models were fitted to data to assess if this might reduce this sensitivity. The compartmental models produced wide-ranging estimates of outcome measures but when fitted to data, the estimates obtained were robust to model structure. This finding suggests that there may be an argument for selecting simple models over complex ones, but the complexity of the model should be determined by the purpose of the model and the use to which it will be put.

## Introduction

Advances in computational power have lead to mathematical modelling being used increasingly to solve real-world problems in all fields and levels of decision making. In epidemiology, mathematical models and in particular compartmental models, have been used to explore (among other things) the emergence and spread of disease, and the impact and efficacy of interventions such as drug treatment, vaccine introduction and parasite control (Murray, 2003). There is no single compartmental model structure that fits all diseases and there are many different structures that may be used to model the same disease. The results of these models are often policy relevant and in many cases used by policy makers to estimate populations at risk, design and implement strategies to combat disease and monitor and evaluate on-going interventions. In this regard, models are and should be subjected to rigorous sensitivity testing (Chubb & Jacobsen, 2010). This testing process involves identifying parameters that strongly influence model outcomes and testing assumptions that when relaxed, strongly influence model results. These model assumptions can pertain to population size and initial conditions among other things. While sensitivity is assessed in the case of model assumptions and parameter values, sensitivity to model structure is not often explored, as the particular model structure is chosen in advance from a suite of models. Studies exploring sensitivity to model structure include Rahmandad & Sterman (2008) who compared compartmental models with agent-based models to assess heterogeneity and network structure and Ferrer *et al* (2012) who evaluated the impact of anti-malarial interventions on compartment and agent-based models. Yet there may still be differences in model predictions between compartmental models of the same disease with equivalent parameters but different model structure. In this regard, this paper explores the sensitivity of a selection of epidemiologically relevant compartmental models that differ only in model structure. While this may seem limited, it serves to show that differences because of model structure may not only occur between classes of models but within them as well. In this paper this sensitivity to model structure is assessed in a number of ways: differences in model predictions in the implementation of routine drug therapy, general vector control (VC), mass drug administration and whether these predictions differ between models if the models have been fitted to data or not. The next section provides an introduction to compartmental models in epidemiology. The models are developed in the section after that while the penultimate section illustrates the results and the final section follows the discussion.

### Compartment models in epidemiology

The first known contribution of mathematical modelling in epidemiology is Daniel Bernoulli’s work on the inoculation against smallpox in 1760 (Brauer et *al*, 2008). Ross, Halmer, Soper, Kermack and McKendrick all contributed to the application of compartmental models to epidemiology between 1900 and 1935 (Anderson & May, 1992). The model proposed by Kermack and McKendrick in 1927 has come to be known as the Susceptible-Infective-Recovered (SIR) model with underlying equations:

(1) $$\frac{dS}{dt}=-\beta SI$$

(2) $$\frac{dI}{dt}=\beta SI-\frac{1}{\alpha }I$$

(3) $$\frac{dR}{dt}=\frac{1}{a}I$$

where *t* is time, S is the susceptible population (at risk of infection), *I* is the infectious population (capable of transmitting infection) and *R* is the recovered population (removed and playing no further role in the epidemic). *β* is the number of contacts per unit time and 1/α is the rate of recovery.

This model results in a fixed population *N (S+I+R)* where members of the population mix homogeneously (interact with one another to the same degree). There is no entry into or departure from the population as the dynamics of the disease are much faster than the time scale of birth and death processes; and hence the impact of these processes on the population can be ignored. Any inherent age, demographic and spatial structure is also ignored. There is no initial immunity as all ‘members’ of the susceptible population are equally likely to get infected. The model infers permanent immunity; once recovered, a second infection is impossible. The incubation period of the infectious agent is instantaneous and the duration of infectivity is the same as the duration of the disease (one is infectious as long as one has the disease). Discrete individuals do not exist in the model and it is assumed that individuals who reside in the compartments are identical and as such variation among individuals is unimportant. Thus compartmental models are described as population-level models. It is fractions of the population that flow between compartments and these movements are continuous. The rate of recovery 1/α is constant for each ‘member’ of the population and hence the average duration of infectiousness (and in this case disease) is *α*.

**Figure 1:**
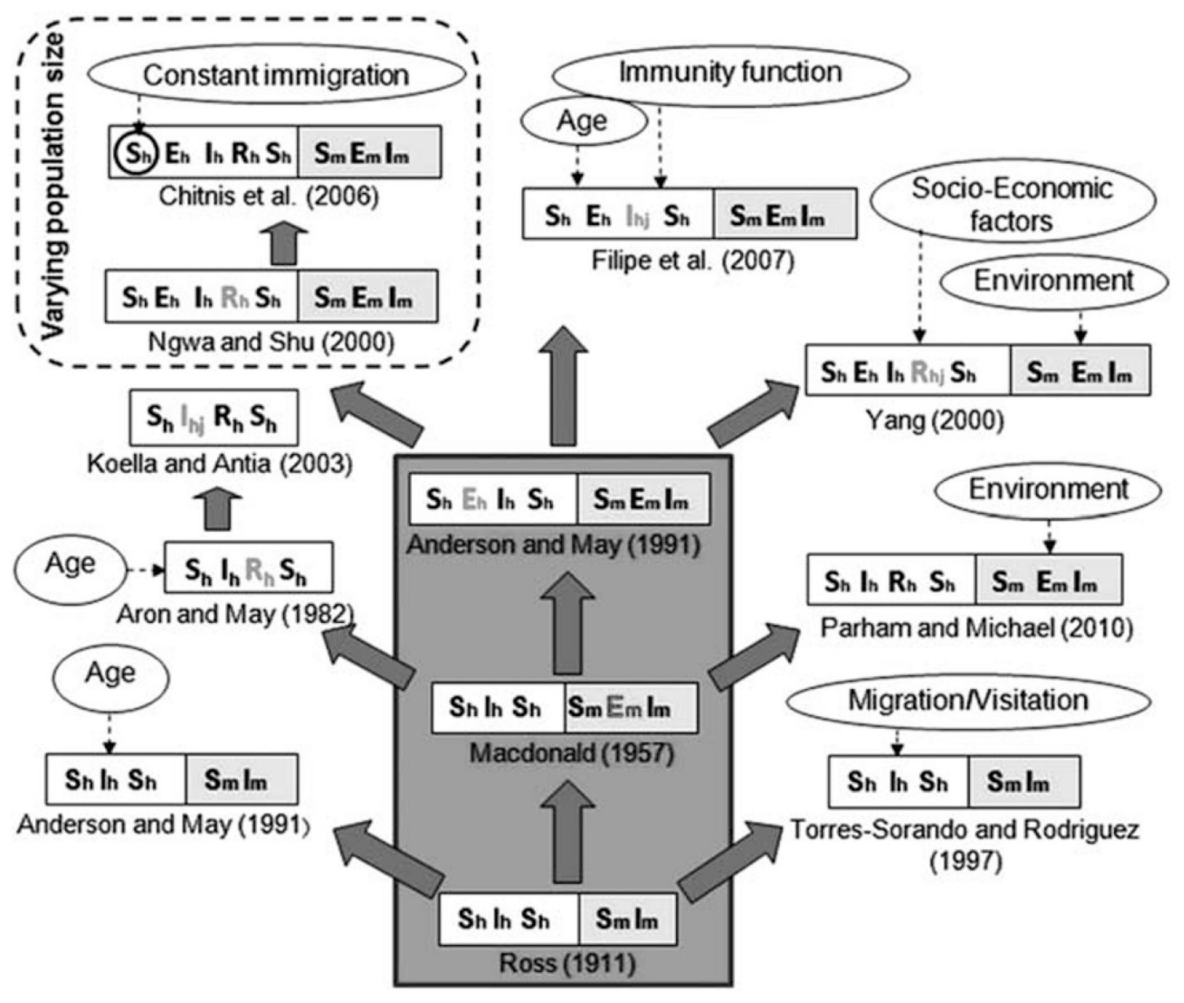
Evolution and grouping of different types of SEIR malaria models. Subscripts ‘h’ and’m’ stands for human and mosquito. Double-folded boxes are for both human & mosquito population, and single fold boxes are only for human. First time addition of a new compartment is shown in grey script. The subscript ‘j’ (= 1, 2, 3) indicates further subdivision of the corresponding compartment. Three models inside the big grey box are considered as the Basic malaria models. Dotted arrows show the incorporation of complex factors in different models or specific compartment (encircled). Total population size is constant for all models, except the ones inside the dashed box. *Source:* Mandal *et al*, (2011): published in Malaria Journal.

There are several extensions of the SIR model, including the Susceptible-Infectious (SI) model where immunity is ignored (by excluding the Recovered compartment), the Susceptible-Exposed-Infectious-Recovered (SEIR) model that allows for a period of latency/exposure before becoming infectious, the Susceptible-Infectious-Recovered-Susceptible (SIRS) model that allows for temporary immunity and other similar models (Jacquez, 1996). As diseases have different characteristics, these models may be extended to include biology (stages of immunity, vector/ pathogen dynamics, super-infection), demography (birth and death processes, age, gender), interventions (drug therapy, vaccines, VC) and geography (spatial structure, migration). Even the same disease may be modelled with very different structures (Figure 1). In the case of malaria, Koella & Antia (2003) published SIRS models incorporating resistance to drug therapy and super-infection, Yang (2000); Yang & Ferreira (2000) incorporated socioeconomic and environmental factors into SEIR models for hosts and vectors (incorporating dynamics in an SEI model for mosquitoes), Torres-Sorando & Rodriguez (1997) included migration and visitation in an SIS model and Auger *et al* (2008) extended the Ross-Macdonald model to several patches in a meta-population compartmental model (Ross, 1911; Mandal *et al*, 2011).

In using several different structures to model the same disease, it is of interest to assess if there are large differences in model predictions because of model structure alone. This paper compares a selection of model structures and the predicted impact of policy interventions. This paper also explores whether fitting the models to data to determine parameter values empirically, reduces sensitivity to model structure.

### Model development

Different model structures are compared for a disease that has a latent period (*L*), a period where clinical symptoms have manifested but the host is not yet infectious (*B*), and an infectious period (*I*) that does not grant immunity that is, a person may be re-infected once susceptible (*S*) again. These models are compared in the absence and presence of drug therapy. Once infected and clinically ill, a patient has the potential to receive drug therapy and those who do not receive drug therapy recover naturally that is, they do not die from the disease but recover through the body’s natural defences at a period longer than the drug recovery period. Patients may seek drug therapy when symptoms have manifested (they feel ill) as well as at the infectious stage. The natural recovery period is assumed to be longer than the drug recovery period and the time to infectiousness, hence natural recovery is only possible once the disease is at the infectious stage and not any earlier. Birth and death, super-infection and the development of immunity through repeated infections are ignored. Such a disease with a latent period and no immunity would need to be modelled using variants of an SEIS general model structure.

**Figure 2:**
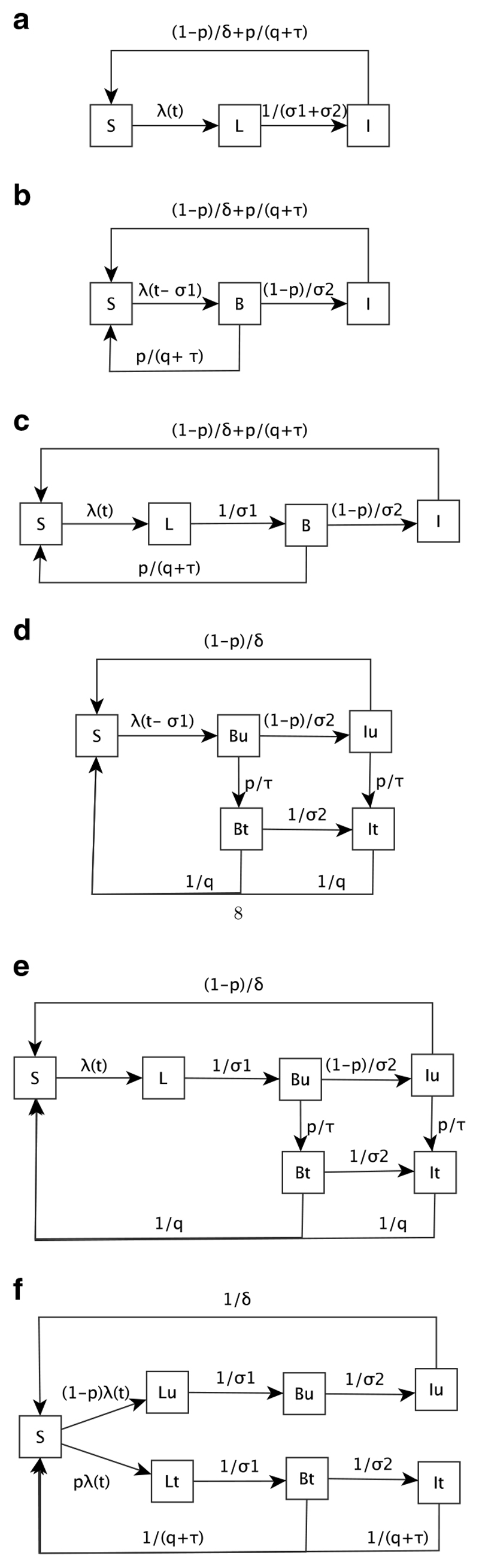
Model flowcharts. (a) Model 1: SLI (With treatment); (b) Model 2: SBI (With treatment); (c) Model 3: SLBI (With treatment); (d) Model 4: Stratified SBI (With treatment); (e) Model 5: Stratified SLBI (With treatment); (f) Model 6: Alternate Stratified SLBI (With treatment).

Three models are compared in the absence of drug therapy (SLI, SBI, SLBI) and five models are compared in the presence of drug therapy (SLI, SBI, SLBI, stratified SBI, and stratified SLBI) (Figure 2). These models are compared with an alternate version of the SLBI model where the probability of receiving treatment is applied *at the time of acquiring infection* rather than during the infection, as an alternative way of capturing the proportion of infections that are treated (Model 6 in Figure 2). This alternate SLBI model assumes a per-infection probability of treatment whereas the other models assume a per unit-time probability of treatment. Differences between models are estimated by measuring incidence and prevalence of the disease and the treatment coverage. This alternate SLBI model is used to simulate data for data-fitting purposes so as to assess if validating models with data through empirical estimation of parameter values reduces the sensitivity of the models to differences in model structure. Finally policy interventions (VC and mass drug administration) are also modelled to assess differences in model predictions because of model structure.

The model parameters driving these models and their assumed values are in Table 1. All models are from the Susceptible-Exposed-Infectious-Susceptible (SEIS) class of models where *L* is used to characterise the latent phase of the disease. In Model 1 (SLI), the latent (*L*) and symptomatic (*B*) stages of the disease have been combined; hence the rate of flow between Latent compartment and the Infectious compartment is 1/(⏃_1_+σ_2_). When incorporating drug therapy into the models, one has to consider at what stage of the disease the population has access to drug therapy. As the disease has a latent stage, it is only in the infectious compartment that the population may seek drug therapy and this occurs at the rate *p/(q+τ)* (incorporating the treatment probability (*p*), the time to seek treatment (*τ*) and the drug recovery period). This rate naturally comprises two steps: treatment seeking and recovery through drug therapy. As there is no treated compartment in this model to explicitly allow for this, the total time to move from being infectious to becoming susceptible again is *q*+*τ* and hence the population who receive drug therapy (with probability *p*) do so at a rate of *p*×1/(*q*+*τ*). The population that is infectious but remains untreated recover naturally at the natural recovery rate ((1–*p*)/δ).

Models 2 (SBI) and 4 (Stratified SBI) do not have a latent compartment, only compartments reflecting the symptomatic (*B*) and infectious *(I)* stages of the disease, where the subscripts *u* and *t* in Model 4 represent untreated and treated infections respectively. These models account for the latent period by including a time delay in the force of infection (*λ*) of size σ_1_. As symptoms have manifested (population feels ill) in the symptomatic compartment (*B*), the population may receive drug therapy from both this and the infectious compartments. At the symptomatic stage, *p*% of the population will seek and receive drug therapy (Model 2) or seek treatment (Model 4) while (1–*p*)% of the population will remain untreated and become infectious. In Model 2, the time to seek treatment is incorporated in the same manner as for Model 1. In Model 4, owing to the inclusion of Symptomatic and Infectious compartments for the *treated* population, the time to seek treatment is incorporated explicitly. The latent and symptomatic stages of the disease are captured separately in Models 3 (SLBI) and 5 (Stratified SLBI) and treatment is incorporated in the same way as Models 2 and 4 respectively. Model 6 stratifies the population into the ‘never treated’ and those ‘destined to be treated’ by multiplying the force of infection *λ* by the treatment probability (*p*). Treatment can take place at both the symptomatic and infectious compartments and occurs at the rate 1/(*q*+*τ*) (incorporating the time to seek treatment and time to drug recovery) while the untreated recover naturally at the natural recovery rate (1/*δ*). The equations underlying all these models are displayed in the Appendices.

Models are compared using steady state measures of incidence, prevalence and treatment coverage. Incidence is measured as the number of new cases at each time step, prevalence is measured as the population infected with the disease (*L, B* or *I*) at each time step and treatment coverage is estimated by cumulative treated cases as a proportion of cumulative cases.

## Results

Models were fitted to data using least squares approach (Hansen *et al*, 2012) and differential equations were solved using the linearised analytic method for ordinary differential equations. All models were programmed in R v3.02 (R Core Group, 2013). Model results are presented under the conditions of no treatment, treatment at different levels, and where external anti-disease interventions are imposed on the models. These results are contrasted between models that have been fitted to data and models that have not.

### No treatment

**Figure 3:**
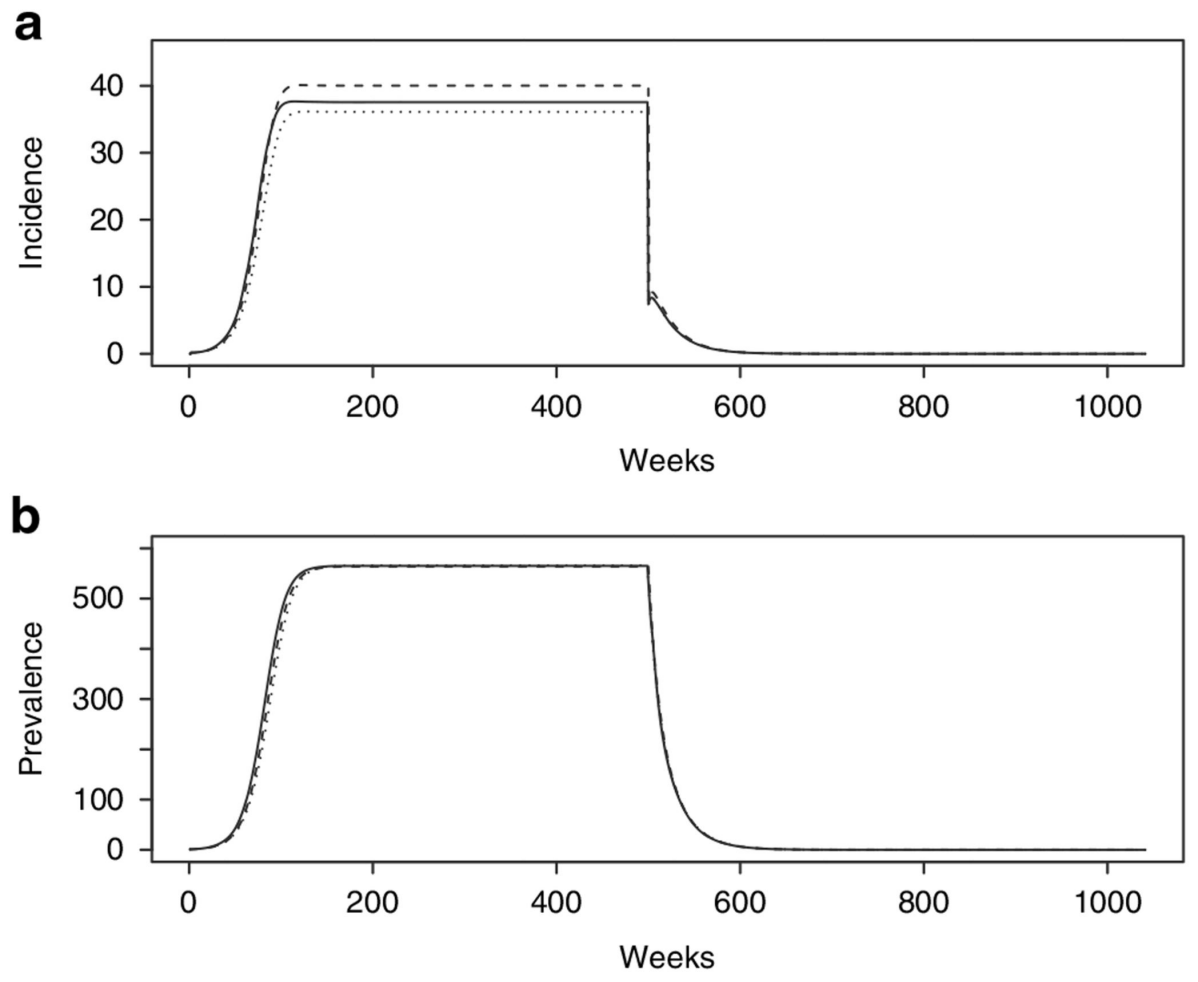
No treatment: Incidence and prevalence for Model 1 (solid), Model 2 (dashed) and Model 3 (dotted).

Of the six models, only models 1, 2 and 3 are tested under the condition of no drug treatment. This is because when the probability of treatment (*p*) is 0, model 4 collapses to model 2 and models 5 and 6 collapse to model 3. Under the condition of no treatment, prevalence in the three models is equivalent while incidence differs slightly among model structures (Figure 3). Incidence in model 2 is higher than models 1 and 3 because the force of infection *λ* is a function of the Infectious compartment and the rate of flow between the Symptomatic and Infectious compartments is faster than that between the Latent and Infectious compartments in model 1. Similarly, incidence in model 3 is lower than in models 1 and 2 as the infectious reservoir is comparatively smaller in model 3.

### With treatment

**Figure 4:**
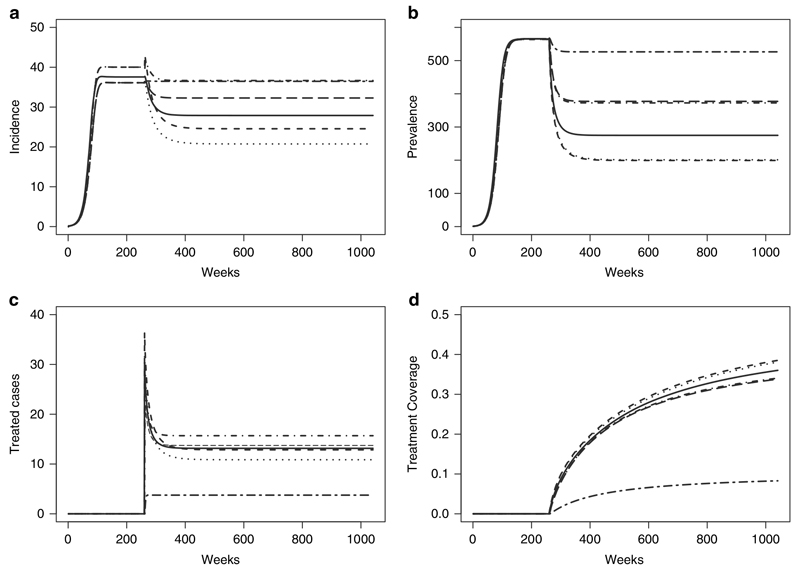
10% Treatment Probability (a) Incidence; (b) Prevalence; (c) Treated Cases and (d) Treatment Coverage for Model 1 (solid), Model 2 (dashed), Model 3 (dotted), Model 4 (dot-dash), Model 5 (long dash) and Model 6 (two dash).

Figure 4 shows that when imposing a 10% treatment probability on the infected population, models 1–5 predict a higher number of treated cases than model 6 and the treatment coverage, as measured by cumulative treated cases as a proportion of cumulative incidence, is estimated correctly by model 6 (10%) but overestimated by the other 5 models. A 10% probability of treatment implies a treatment coverage between 34 and 39% in models 1–5. The two stratified models (4 and 5) behave similarly in that they have the same prevalence and treatment coverage. Likewise models 2 and 3 behave similarly. The treatment coverage is higher in all models compared with the model 6 because these models predict a higher number of treated cases and lower incidence. At a 50% treatment probability models 1–5 predict a lower number of treated cases than model 6 and the treatment coverage is estimated correctly by model 6 (50%) but underestimated by the other five models (10.6-17.2%). This is because Models 1–5 also predict that incidence and prevalence will decrease to 0, albeit at different rates, while model 6 maintains stable non-zero prevalence and incidence levels. At a 100% treatment probability, all models predict a decrease in incidence and prevalence to 0, again at varying rates, with model 6 decreasing to 0 at the slowest rate compared to the other models. These results are shown in the Appendices.

### Treatment coverage and the probability of treatment

The results in the section ‘With treatment’ show large differences in treatment coverage in all six models. It is only in model 6 that the treatment coverage reflects the treatment probability. Exploring why this may be so requires understanding of how treatment coverage is estimated mathematically. Generally values of the parameters that drive compartmental models may be sourced from literature, take on assumed values or may be estimated from data. In modelling the impact of drug therapy, the proportion of infections that are treated (treatment coverage in this paper) is sometimes the best source of data available to mathematical modellers to use to estimate the treatment probability (the chance of receiving drug therapy) in their models. As shown in the results, depending on the structure of the compartmental model, these two values (treatment coverage and the treatment probability) will not always be equal. Treatment probability *p* is *used in the models* to determine the proportion of the infected and infectious populations that get treated and treatment coverage is *estimated from the models* to determine cumulatively, the proportion of cases that actually get treated.

In order to assess the relationship between treatment probability (p) and treatment coverage (denoted as *π*), consider a simple SIS model *(S+I =* 1) for a cohort of infected individuals governed by the equation:

(4) $$\frac{dI}{dt}=\beta IS-\left(\frac{p}{q}+\frac{1-p}{\delta }\right)I$$

where *β* is the number of contacts per unit time, *p* is the probability of treatment, 1/*q* is the drug recovery rate and 1/*δ* is natural recovery rate. For ease of computation let *a* = *p*/*q*+(1–*p*)/*δ* and assume initial conditions of S(0) = 0 and I(0) = 1.

Using the result that in an SIS model, *S*= 1–*I*,

(5) $$\frac{dt}{dt}=\beta I(1-I)-aI.$$

Solving this differential equation amounts to solving,

(6) $$\int_{I(0)}^{I(t)}\frac{dI}{\beta I(1-I)-aI}=\int_{0}^{t}ds.$$

Using the initial condition *I*(0) = 1, this integrates to

(7) $$-\left(\frac{\ln(I)-ln(a+\beta I-\beta )}{a-\beta }-\frac{ln(a)}{a-\beta }\right)=t$$

and hence

(8) $$I=\frac{(a-\beta )e^{(\beta -a)t}}{a-\beta e^{(\beta -a)t}}.$$

The treatment coverage (*π*) is a function of the cumulative treated cases (*pl/q*) and the cumulative cases (*β*IS).

In particular,

(9) $$\pi =\underset{t\to \infty }{\lim}\frac{{\int^{\text{​}}}_{0}^{t}\frac{p}{q}Idt}{{\int^{\text{​}}}_{0}^{t}\beta I(1-I)dt}$$

(10) $$=\underset{t\to \infty }{\lim}\frac{{\int^{\text{​}}}_{0}^{t}\frac{2}{g}\left(\frac{(a-\beta )e^{(\beta -a)t}}{a-\beta e^{\beta -a)t}}\right)dt}{{\int^{\text{​}}}_{0}^{t}\beta \left(\frac{(a-\beta )e^{(\beta -a)t}}{a-\beta e^{\beta -a)t}}\right)\left(1-\frac{(a-\beta )e^{(\beta -a)t}}{a-\beta e^{\beta -a)t}}\right)dt}$$

(11) $$=\underset{t\to \infty }{\lim}\frac{\frac{p}{a\beta }\left(ln\left(\beta e^{\beta t}-ae^{at}\right)-at-ln(\beta -a)\right)}{\frac{-a(a-\beta )}{\beta }\left(\frac{\beta e^{e}}{a\left(\beta e^{a}-ae^{ait}\right)}+\frac{at}{a-\beta }+\frac{\ln\left(\beta e^{e}-ae^{at}\right)}{\beta -a}-\frac{\beta }{a(\beta -a)}-\frac{\ln(\beta -a)}{\beta -a}\right)}.$$

Using L’Hospital’s rule to simplify the limit equation (L’Hospital, 1696):

(12) $$\pi =\underset{t\to \infty }{\lim}\frac{\frac{p}{q\beta }\left(\frac{\beta (a-\beta )e^{bt}}{ae^{at}-\beta e^{\beta t}}\right)}{\frac{-a(a-\beta )}{\beta }\left(\frac{\beta^{2}e^{\beta t}\left(e^{\beta t}-e^{at}\right)}{{\left(ae^{at}-\beta e^{\beta t}\right)}^{2}}\right)}$$

(13) $$=\underset{t\to \infty }{\lim}\frac{p}{-qa(a-\beta )}\times \frac{a-\beta }{\beta }\left(\frac{ae^{at}-\beta e^{\beta t}}{e^{\beta t}-e^{at}}\right).$$

Eq. (13) will tend to a finite limit depending on the relation between *a* (the average recovery rate) and *β* (the transmission coefficient). To establish this relation, one can look at the dynamics of the disease when the SIS model has reached a steady state. At equilibrium, the sum of the inflows to a compartment is equal to the sum of its outflows. Thus for the infectious compartment (*I*) in an SIS model,

(14) $$\beta IS=\left(\frac{p}{q}+\frac{1-p}{\delta }\right)I$$

Substituting *a* = *p/q*+(1−*p*)/*δ* and *S* = 1−*I* into Eq. (14) leads to

(15) βI(1−I)=aI

This simplifies to

(16) $$I=\frac{\beta -a}{\beta }.$$

This system has two equilibria:

1. The trivial (infection-free) equilibrium
2. The non-trivial equilibrium where infection is present

Both equilibria exist depending on the relationship between *β* and a. If *β*<*a*, the trivial equilibrium is stable but if *β* > *a* then the non-trivial equilibrium, the equilibrium where infection is present is non-negative and stable.

Including *β* > *a* and multiplying through by *e*^−*βt*^/*e*^−*βt*^ leads to

(17) $$\pi =\underset{t\to \infty }{\lim}\frac{p}{-qa(a-\beta )}\times \frac{a-\beta }{\beta }\left(\frac{ae^{(a-\beta )t}-\beta }{1-e^{(a-\beta )t}}\right)$$

(18) $$=\frac{p}{-qa(a-\beta )}\times \frac{a-\beta }{\beta }\times (-\beta )$$

(19) $$=\frac{p}{qa}$$

(20) $$=\frac{\frac{p}{q}}{\left(\frac{p}{q}+\frac{1-p}{\delta }\right)}.$$

Thus for an SIS model, treatment coverage (π) is not equal to the treatment probability (*p*), but rather a function of the *p, δ* and *q*.

This result holds for Model 1 but will change for models with different structures (Models 2–5). The results in the section ‘With treatment’ showed that treatment coverage in Model 6 correctly represented the 10% treatment probability. This is because at equilibrium in Model 6,

(21) $$p\lambda S=\frac{1}{\sigma_{1}}L$$

(22) $$\frac{1}{\sigma_{1}}L=\left(\frac{1}{\sigma_{2}}+\frac{1}{q+\tau }\right)B$$

(23) $$\frac{1}{\sigma_{2}}B=\frac{1}{q+\tau }I.$$

Therefore,

(24) $$p\lambda S=\frac{1}{q+\tau }(B+I)$$

and,

(25) $$p=\frac{\frac{1}{q+\tau }(B+I)}{\lambda S}.$$

Treatment coverage (*π*) at equilibrium is treated cases ((*B*+*I*)/(*q*+*τ*)) as a proportion of incidence (*λS*) and hence for Model 6, *π*=*p*. Thus while treatment coverage data is often used by modellers as the best estimate of treatment probability, depending on the model structure, the treatment coverage predicted by the model may be very different from the treatment probability.

### Data fitting

Showing that for different model structures, treatment coverage is not always equal to treatment probability, demonstrates that even for routine anti-disease interventions like drug therapy, model structure has a large impact on model results. This section explores if fitting models to data reduces this sensitivity to model structure that is, if models are fitted to data, does model structure matter less or at all? Model 6 is used to simulate the number of treated cases for data fitting purposes as this was the only one of the six models where *p* = *π*. Many national health systems collect routine data on cases that are treated so this would be typical of data available to mathematical modellers. It is not usually the case that true incidence data would be available as the untreated infected population is generally hidden from the health system. The treated cases from all five models are fitted to the model 6 data estimating the value of *p* (0.1 in the model 6). All other parameter values are held constant at their assumed values in Table 1. Parameter estimation is achieved using the Least Squares algorithm for data fitting. All five models fit the data well with the estimated value of *p*, the probability of being treated, being in the range of 0.0063–0.0097. At these values of *p*, the six models make identical predictions for prevalence, treated cases and treatment coverage with only small differences observed in the prediction of incidence, thereby showing the decrease in sensitivity to model structure that can be achieved by fitting models to data.

### Impact of other interventions

Compartment and other models of disease transmission may be used to assess the impact of policy interventions on transmission. Two interventions are applied to the six models to assess sensitivity because of model structure; mass drug administration and VC. These interventions are then scaled up, aimed at eliminating the disease.

#### Mass drug administration

**Figure 5:**
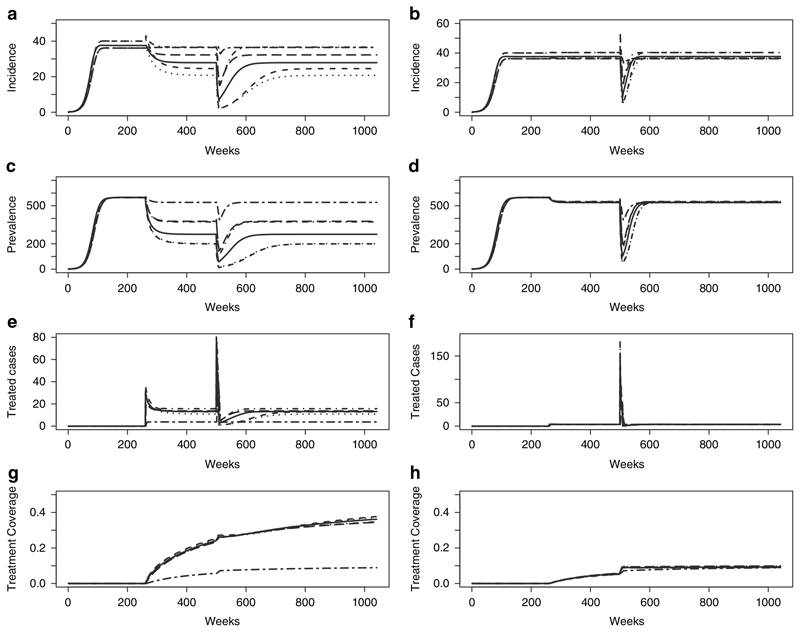
Mass Drug Administration (8 weeks, 75% probability of treatment). (a & b) Incidence; (c & d) Prevalence; (e & f) Treated Cases and (g & h) Treatment Coverage for Model 1(solid), Model 2 (dashed), Model 3 (dotted), Model 4 (dot-dash), Model 5 (long dash) and Model 6 (two dash).

Mass drug administration involves administering drug therapy to the population at risk regardless of disease status (Greenwood, 2010). An example of this intervention is reflected in the models by an increase in the treatment probability *p*. Mass drug administration is implemented over 8 weeks at 75% probability in all models. The graphs on the left in Figure 5 show the impact of this intervention on models that have not been fitted to data while the graphs on the right show the impact this intervention has on the models once they were fitted to the data from model 6. The results show that when mass drug administration is applied to the fitted models, all models reach the same equilibrium before the intervention and show an increase in the number treated at the time of the intervention but revert to the previous equilibrium eventually, whereas models that were not fitted to data reached different points of equilibrium. Even though the fitted models achieve the same pre- and post-intervention equilibrium, the immediate impact (decrease in incidence and prevalence) of the intervention varies between model structures.

#### Vector control

**Figure 6:**
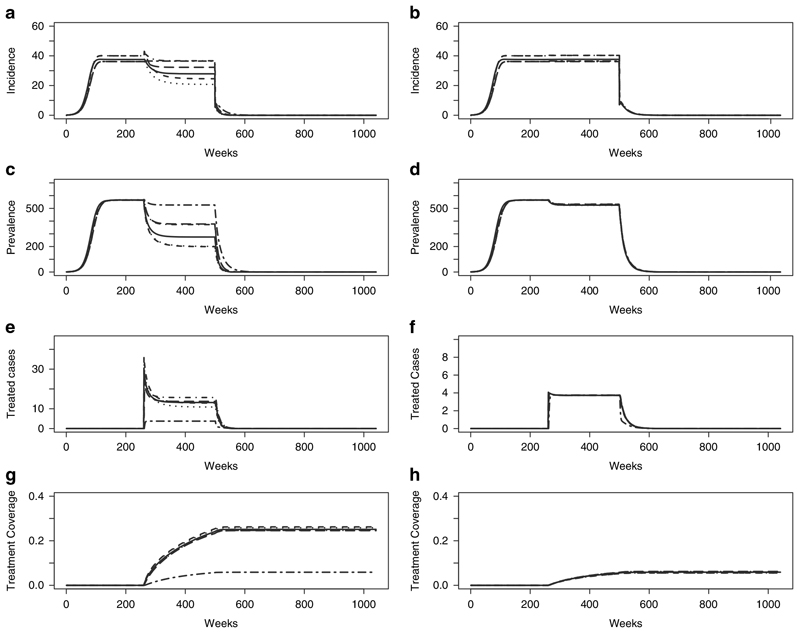
Vector control (50% Coverage, 50% Efficacy) (a & b) Incidence; (c & d) Prevalence; (e & f) Treated Cases and (g & h) Treatment Coverage for Model 1 (solid), Model 2 (dashed), Model 3 (dotted), Model 4 (dot-dash), Model 5 (long dash) and Model 6 (two dash).

In some diseases like malaria and dengue, vectors are an active part of disease transmission. VC as an intervention, requires acting on the vector population in a way that interrupts transmission. This may for example be achieved through larviciding, the use of insecticide treated bednets and household spraying with pesticides (World Health Organization, 1997). In models 1–6, this intervention is captured as having a decreasing effect on *β;* the number of contacts with vectors. The coverage of the control as well as the efficacy of process are taken into account in decreasing *β* where *β_VC_* = (1–cover-age*efficacy)*β. In these models an example of VC is introduced into the models with 50% coverage and 50% efficacy. Figure 6 (left) shows that the different model structures (not fitted to data) measure the impact of VC very differently; some showing a much greater impact on prevalence and incidence than others. Once the models have been fitted to data however, these differences are minimal (Figure 6 (right)).

#### Modelling to disease elimination

When interventions are used to eliminate a disease rather than attempt to control it, mathematical models will aim to produce estimates of the length of time to eliminate the disease, the intensity of each of intervention as well as the combinations of interventions that may be used to eliminate a disease. Elimination of infection is defined as the reduction to 0 of locally acquired incidence in a geographical area as the result of deliberate efforts (Dowdle, 1998). Two very resource-intensive interventions aimed at eliminating a disease are tested on the six models: VC with a 90% coverage and at a 90% efficacy and a scaling up of drug therapy from a 10% treatment probability to a 90% treatment probability. As the compartments and hence the flows between compartments will never actually reach 0 to achieve elimination as outlined above, the disease is assumed to be eliminated when prevalence decreases below 10^−6^. The impact of these interventions is estimated as the time to elimination (in weeks) from the start of the interventions. These interventions are tested on the six models that have not been fitted to data and on the models once they have been fitted to data.

Table 2 shows that by scaling up VC, the models that are not fitted to data predict widely varying time to elimination values ranging from 193 weeks (<4 years) to 458 weeks (<9 years). When fitted to data the predictions fall in a narrower range between 416 and 458 weeks. The results are quite different for a drug scale up intervention. When fitting the models to data, *p*, the probability of treatment, is the estimated parameter from the data-fitting process. Thus when uniformly increasing the *p* to 0.9 at the start of the intervention, the previously fitted value falls away and hence the time to elimination estimates are the same regardless of whether the models are fitted or not. These estimates range widely from 50 weeks (<1 year) to 399 weeks (<8 years) depending on the model structure. These estimates are not robust and clearly sensitive to model structure.

## Discussion

As mathematical modelling is increasingly used to aid decision making in the public health sector models need to be rigorously tested for sensitivity to parameter values and model assumptions so that model predictions are robust (Chubb & Jacobsen, 2010). Incorporating the standard intervention of drug therapy produced very different results for all models tested in terms of prevalence, incidence and the treatment coverage. With only a 10% treatment probability, the decrease in prevalence of the disease ranges between 3 and 49%. At higher treatment rates (> 50%), the disease was even eliminated in some models. These differences occurred for models with the same parameter values and model assumptions but different model structures. The practical significance of these differences is great in that reducing a disease marginally (3%) or reducing a disease by half have very different impacts on a public health sector with minimal scarce resources and high opportunity cost on these resources. This is reiterated by groups such as the Mal-ERA Consultative Group on Modelling, who recognised the contribution modelling can make to the elimination of malaria globally and developed a framework of priority areas for modelling to inform such as optimal resource allocation and expected timelines to achieve goals (The malERA Consultative Group on Modeling, 2011). Model results need to be robust to model structure if they are to play a role in informing strategy and policy design, where scarce resources will be committed on the basis of the models’ predictions.

Fitting the five models to data simulated from model 6 (to empirically estimate the treatment probability), shows that the sensitivity because of model structure can be reduced. As countries become better at collecting routine data and performing clinical trials, opportunities exist for modellers to validate their models with real data. Data quality issues aside, modellers may still be able to use their models to reproduce the data, as well as validate model predictions with a testing dataset. As models are extended to incorporate specific disease dynamics such as immunity (Aron, 1988; Yang & Ferreira, 2000; Smith *et al*, 2006), vector populations (Dietz, 1980; Luz *et al*, 2010) and geography (Brauer, 2001; Juan, 2006; Tumwiine *et al*, 2010), different model structures may well produce very different results and predictions. Fitting models to data where possible can reduce the sensitivity of the model results to model structure.

In attempting to eliminate the disease using VC, it was found that fitting the models to data decreased the sensitivity to model structure, but this was not the case for drug scale-up. The varying predictions on time to eliminate the disease can be partly explained by the treatment seeking behaviour defined in each model. In model 1, the population only has one chance at receiving drug therapy (Infectious compartment), whereas in models 2 and 3, the population has two chances to receive drug therapy (Symptomatic and Infectious compartments) and hence it is harder to eliminate the disease in model 1 than in models 2 and 3. The longest time to elimination estimate is from model 6 (the alternate stratified SLBI). In this model, a segment of the population has no chance to receive drug therapy in the course of the infection and will have to wait until the next round of infections to stand a chance of receiving drug therapy. In this case, it is much harder to eliminate the disease. Modellers should be aware of the treatmentseeking behaviour of the populations they model, but where this information is not available, there is an argument for modelling different treatment-seeking behaviours so that this heterogeneity may be better understood and prudent and conservative estimates may be produced. O’Connell *et al* (2012) found in their study of malaria in Cambodia that treatment-seeking behaviour is complex, often driven by cultural norms and other practicalities.

Some of the models in this paper have a simple structure while others have more complex structures. The results have shown that models seeking to measure the same phenomenon (e.g. estimating the impact of drug therapy) that differ in structure only, can produce very similar results *if they are fitted to the same set of data*. Does the argument exist for choosing simple models over complex ones? The answer is yes, but the converse is also true. On one extreme one could have a model that is too simple to be biologically plausible and on the other extreme, the most biologically plausible model may be too complex to be of any practical use. There needs to be a balance between model simplicity and biological plausibility for models to be of practical relevance. White *et al* (2009) compared the results of a simple deterministic compartmental model with other agent-based stochastic complex models in a malariaelimination context. They concluded that in situations where data is sparse, yet urgency exists to provide input into strategy design, simple model structures are suitable but complex models can provide more information (on the context being modelled), especially in the long term.

This paper sought to compare results between deterministic compartmental models only and did not extend to include other model structures such as agent-based models and time series models. While this is a limitation of the paper, it also serves to show that differences in results because of model structure arise not only among different classes of models but among models in the *same* class also. Several aspects have been ignored such as immunity, super infection, chemoprophylaxis, drug resistance, heterogeneity of the population and vector population dynamics. Stochasticity was also ignored. It was not in the scope of the paper to include all aspects of disease, but rather to show that even in the most basic disease context, differences still exist because of model structure. These are all areas where further work can be undertaken. Ultimately, model structure (and its complexity) needs to be chosen carefully depending on the focus of the model and the use to which it will be put.

Compartmental model structures are based on the underlying epidemiological and demographic interactions of a particular disease. Given that there are many choices for these interactions, the number of possible combinations are large (Hethcote, 1994). This manuscript is one of few to compare a selection of these models to assess their similarities and differences. The primary contribution of this paper is to show that not all models are equal. Differences exist even with the smallest changes in model structure and increases in complexity of models may result in different conclusions being drawn from the model predictions. Gaining a clear understanding of these models enables one to choose a structure that is suitable to the epidemiological process being modelled. Additionally the paper shows that fitting models to data can reduce the differences because of model structure, but even so offers limited assistance once models are used for predictions. One purpose of a mathematical model is to simulate a current epidemiological situation for the purposes of testing potential policy interventions to assess their impact on the disease. This often requires expanding on the existing model structure to accommodate these interventions. As interventions may be modelled in many ways, differences in model predictions may still result because of differences in structure only. This is often not considered. Fitting models to data will assist with simulating the current epidemiological situation, but not with the predictions on the proposed policy interventions. These model predictions can be made robust by introducing stochasticity into the model to produce a range of plausible predictions. Second, the interventions themselves should be modelled in a set of different structures to assess the range in predictions and gain an understanding of the impact that the chosen model structure has on the output. Ultimately, mathematical models will not all produce the same results and thus an in depth understanding of the model structure is necessary to maximise the usefulness of these models.

## Supplementary Material

Appendices

## Figures and Tables

**Table 1:** Model parameters: Description and value

Parameter	Description	Value	Rate of flow
σ_1_	Period of latency	1 week	1/σ_1_
σ_2_	Time to infectiousness	1 week	1/σ_2_
*τ*	Time to seek treatment	0.5 week	1/τ
*q*	Drug recovery period	1 week	1/q
*-*	Natural recovery period	12 weeks	1/δ
*p*	Probability of treatment	0, 0.1, 0.5, 1	
*N*	Population Size	1000 people	
*β*	Contact Rate	10 per annum	*β*
λ	Force of Infection	*β×I/N(Models* 1,2&3) *β*×(I_u_+I_t_)/*N*(Models 4, 5&6)	

**Table 2:** Time to elimination (weeks) for vector control and scale-up of drug therapy

Time to Elimination (weeks)	Vector Control			Drug Scale-up	
	Not Fitted	Fitted		Not Fitted	Fitted
Model 1	206	416		120	120
Model 2	193	424		50	50
Model 3	197	426		54	54
Model 4	260	432		121	121
Model 5	266	434		241	241
Model 6	458	458		399	399
