# Supplementary material for: Sensitivity to model structure: a comparison of compartmental models in epidemiology
Source: Health Syst (Basingstoke). Author manuscript; Available in PMC 2022 Sep 2. (PMC7613485; doi:10.1057/hs.2015.2)
Supplement: Appendices [file EMS153224-supplement-Appendices.pdf]

MURRAY JD (2003) *Mathematical Biology II*, Volume 18. Springer, Berlin. ISBN 0387952284. [WWW document] http://www.amazon.com/Mathematical-Biology-II-J-D-Murray/dp/0387952284.

O'CONNELL KA *et al* (2012) Souls of the ancestor that knock us out and other tales. A qualitative study to identify demand-side factors influencing malaria case management in Cambodia. *Malaria journal*, 11(1), 335.

RAHMANDAD H and STERMAN J (2008) Heterogeneity and network Structure in the dynamics of diffusion: Comparing agent-based and differential equation models. *Management Science*, 54(5), 998–1014.

ROSS R (1911) *The prevention of Malaria*. John Murray, Albemarle Street, London.

R Core Group (2013) [WWW document] www.r-project.org (accessed 19 August 2013).

SMITH T *et al* (2006) Mathe-matical modeling of the impact of malaria vaccines on the clinical epidemiology and natural history of *Plasmodium falciparum* malaria: Overview. *The American Journal of Tropical Medicine and Hygiene* **75(suppl 2)**, 1–10.

The malERA Consultative Group on Modeling (2011) A research agenda for malaria eradication: modeling. *PLoS medicine*, 8(1), January. ISSN 1549-1676. doi: 10.1371/journal.pmed.1000403. [WWW document] http://journals.plos.org/plosmedicine/article?id=10.1371/journal.pmed.1000403.

TORRES-SORANDO L and RODRIGUEZ DJ (1997) Models of spatio-temporal dynamics in malaria. *Ecological Modelling*, 104(2–3), 231–240.

TUMWIINE J, MUGISHA JYT and LUBOOBI LS (2010) A host-vector model for malaria with infective immigrants. *Journal of Mathematical Analysis and Applications*, 361 (1), 139–149.

World Health Organization (1997) Vector control: methods for use by individuals and communities. Technical report. [WWW document] http://www.who.int/malaria/publications/atoz/9241544945/en/index.html (accessed 19 August 2013).

WHITE LJ *et al* (2009) The role of simple mathematical models in malaria elimination strategy design. *Malaria Journal*, 8(8), 212.

YANG HM (2000) Malaria transmission model for different levels of acquired immunity and temperature-dependent parameters (vector). *Revista de saude publica*, 34(3), 223–231.

YANG HM and FERREIRA MU (2000) Assessing the effects of global warming and local social and economic conditions on the malaria transmission. *Revista de saude publica*, 34(3), 214–222.

Appendix A

Model equations

Model 1 (SLI)

$$\frac{dS}{dt} = -\lambda_t S + \frac{1-p}{\delta} I + \frac{p}{q+\tau} I$$

$$\frac{dL}{dt} = \lambda_t S - \frac{1}{\sigma_1+\sigma_2} L$$

$$\frac{dI}{dt} = \frac{1}{\sigma_1+\sigma_2} L - \frac{1-p}{\delta} I - \frac{p}{q+\tau} I$$

Model 2 (SBI)

$$\frac{dS}{dt} = -\lambda_{(t-\sigma_1)} S + \frac{1-p}{\delta} I + \frac{p}{q+\tau}(B+I)$$

$$\frac{dB}{dt} = \lambda_{(t-\sigma_1)} S - \frac{1-p}{\sigma_2} B - \frac{p}{q+\tau} B$$

$$\frac{dI}{dt} = \frac{1-p}{\sigma_2} B - \frac{1-p}{\delta} I - \frac{p}{q+\tau} I$$

Model 3 (SLBI)

$$\frac{dS}{dt} = -\lambda_t S + \frac{1-p}{\delta} I + \frac{p}{q+\tau}(B+I)$$

$$\frac{dL}{dt} = \lambda_t S - \frac{1}{\sigma_1} L$$

$$\frac{dB}{dt} = \frac{1}{\sigma_1} L - \frac{1-p}{\sigma_2} B - \frac{p}{q+\tau} B$$

$$\frac{dI}{dt} = \frac{1-p}{\sigma_2} B - \frac{1-p}{\delta} I - \frac{p}{q+\tau} I$$

Model 4 (Stratified SBI)

$$\frac{dS}{dt} = -\lambda_{(t-\sigma_1)} S + \frac{1-p}{\delta} I_U + \frac{1}{q}(B_T + I_T)$$

$$\frac{dB_U}{dt} = \lambda_{(t-\sigma_1)} S - \frac{1-p}{\sigma_2} B_U - \frac{p}{\tau} B_U$$

$$\frac{dI_U}{dt} = \frac{1-p}{\sigma_2} B_U - \frac{1-p}{\delta} I_U - \frac{p}{\tau} I_U$$

$$\frac{dB_T}{dt} = \frac{p}{\tau} B_U - \frac{1}{\sigma_2} B_T - \frac{1}{q} B_T$$

$$\frac{dI_T}{dt} = \frac{p}{\tau} I_U + \frac{1}{\sigma_2} B_T - \frac{1}{q} I_T$$

Model 5 (Stratified SLBI)

$$\frac{dS}{dt} = -\lambda_t S + \frac{1-p}{\delta} I_U + \frac{1}{q}(B_T + I_T)$$

$$\frac{dL}{dt} = \lambda_t S - \frac{1}{\sigma_1} L$$

$$\frac{dB_U}{dt} = \frac{1}{\sigma_1} L - \frac{1-p}{\sigma_2} B_U - \frac{p}{\tau} B_U$$

$$\frac{dI_U}{dt} = \frac{1-p}{\sigma_2} B_U - \frac{1-p}{\delta} I_U - \frac{p}{\tau} I_U$$

$$\frac{dB_T}{dt} = \frac{p}{\tau} B_U - \frac{1}{\sigma_2} B_T - \frac{1}{q} B_T$$

$$\frac{dI_T}{dt} = \frac{p}{\tau} I_U + \frac{1}{\sigma_2} B_T - \frac{1}{q} I_T$$

Model 6 (Alternate Stratified SLBI)

$$\frac{dS}{dt} = -\lambda_t S + \frac{1}{\delta} I_U + \frac{1}{q+\tau}(B_T + I_T)$$
$$\frac{dL_U}{dt} = (1-p)\lambda_t S - \frac{1}{\sigma_1} L_U$$
$$\frac{dB_U}{dt} = \frac{1}{\sigma_1} L_U - \frac{1}{\sigma_2} B_U$$
$$\frac{dI_U}{dt} = \frac{1}{\sigma_2} B_U - \frac{1}{\delta} I_U$$
$$\frac{dL_T}{dt} = p\lambda_t S - \frac{1}{\sigma_1} L_T$$
$$\frac{dB_T}{dt} = \frac{1}{\sigma_1} L_T - \frac{1}{\sigma_2} B_T - \frac{1}{q+\tau} B_T$$
$$\frac{dI_T}{dt} = \frac{1}{\sigma_2} B_T - \frac{1}{q+\tau} I_T$$

Appendix B

Additional output

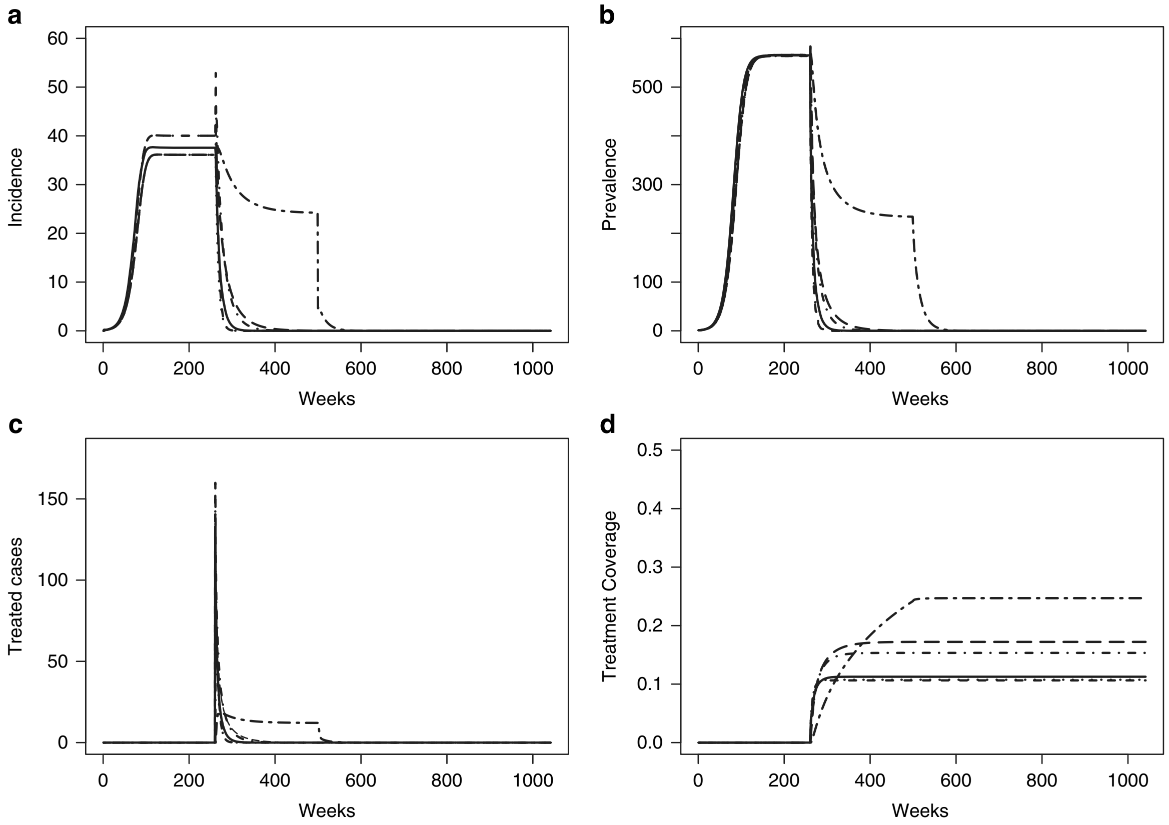

Figure B1 50% Treatment Probability. (a) Incidence; (b) Prevalence; (c) Treated Cases and (d) Treatment Coverage for Model 1 (solid), Model 2 (dashed), Model 3 (dotted), Model 4 (dot-dash), Model 5 (long dash) and Model 6 (two dash).
